# Molecular Mechanisms Involved in the Chemical Instability of ONC201 and Methods to Counter Its Degradation in Solution

**DOI:** 10.3390/pharmaceutics15102371

**Published:** 2023-09-22

**Authors:** Maxime Annereau, Marina Vignes, Lucas Denis, André Rieutord, François-Xavier Legrand, François Rioblanc, Muriel Paul, Jacques Grill, Philippe-Henri Secretan, Bernard Do

**Affiliations:** 1 Université Paris-Saclay, 91400 Orsay, France; maxime.annereau@gustaveroussy.fr (M.A.); marina.vignes@gustaveroussy.fr (M.V.); bernard.do@aphp.fr (B.D.); 2 Clinical Pharmacy Department, Gustave Roussy Cancer Campus, 94800 Villejuif, France; lucas.denis@gustaveroussy.fr (L.D.); andre.rieutord@gustaveroussy.fr (A.R.); francois.rioblanc@gustaveroussy.fr (F.R.); 3 Université Paris-Saclay, CNRS, Institut Galien Paris-Saclay, 91400 Orsay, France; francois-xavier.legrand@universite-paris-saclay.fr; 4 Department of Pharmacy, Henri Mondor Hospital, AP-HP, 94000 Creteil, France; muriel.paul@aphp.fr; 5 EpidermE, Université Paris Est Creteil, 94000 Creteil, France; 6 Molecular Predictors and New Targets in Oncology, INSERM, Gustave Roussy, Université Paris-Saclay, 94800 Villejuif, France; jacques.grill@gustaveroussy.fr; 7 Département de Cancérologie de l’Enfant et de l’Adolescent, Gustave Roussy, Université Paris-Saclay, 94800 Villejuif, France; 8 Université Paris-Saclay, Matériaux et Santé, 91400 Orsay, France

**Keywords:** ONC201, oxidation mechanism, photolysis mechanism, DFT, antioxidant effects, preformulation studies

## Abstract

Glioblastoma is one of the most common and aggressive forms of brain tumor, a rare disease for which there is a great need for innovative therapies. ONC201, a new drug substance, has been used in a compassionate treatment program where the choice of dosage form and regimen have yet to be justified. The prior knowledge needed to anticipate ONC201 stability problems has recently been partially addressed, by (i) showing that ONC201 is sensitive to light and oxidation and (ii) identifying the molecular structures of the main degradation products formed. The aim of the work presented here was to improve our understanding of the degradation pathways of ONC201 using data from ab initio calculations and experimental work to supplement the structural information we already published. The C–H bonds located αto the amine of the tetrahydropyridine group and those located *alpha* to the imine function of the dihydroimidazole group exhibit the lowest bond dissociation energies (BDEs) within the ONC201 molecule. Moreover, these values drop well below 90 kcal.mol^−1^ when ONC201 is in an excited state (S1; T1). The structures of the photoproducts we had previously identified are consistent with these data, showing that they would have resulted from radical processes following the abstraction of *alpha* hydrogens. Concerning ONC201’s sensitivity to oxidation, the structures of the oxidation products matched the critical points revealed through mapped electrostatic potential (MEP) and average local ionization energy (ALIE). The data obtained from ab initio calculations and experimental work showed that the reactivity of ONC201 to light and oxidation conditions is highly dependent on pH. While an acidic environment (pH < 6) contributes to making ONC201 quantitatively more stable in solution in the face of oxidation and photo-oxidation, it nevertheless seems that certain chemical groups in the molecule are more exposed to nucleophilic attacks, which explains the variation observed in the profile of degradation products formed in the presence of certain antioxidants tested. This information is crucial to better understand the stability results in the presence of antioxidant agents and to determine the right conditions for them to act.

## 1. Introduction

ONC201 (dordaviprone) is a new drug currently being evaluated to improve the therapeutic management of glioblastoma [1]. To date, it is used in France in a compassionate program approved by the French medical authorities [2].

In charge of its pharmaceutical development, our work involves improving our knowledge of the intrinsic stability profile of ONC201, a molecule previously unknown in the pharmaceutical field.

Previously, we had written a chapter as part of a program to study the pre-formulation of ONC201. The aim of this first part of the work was to gain access to the structure of the main degradation products of ONC201 formed following its exposure to stress conditions [3]. The degradation products identified were thus described for the first time, bearing in mind that they only appeared under conditions of oxidative and photolytic stress. The molecule is in fact resistant to thermal and hydrolytic conditions. Based on the nature of the resulting degradation products, we found that, in both cases, structural changes occurred mainly in the imidazole, tetrahydropyridine and/or piperidine functions of the molecule, involving in some cases the displacement of benzyl and o-xylene groups. In this first approach, prior knowledge of the structures of the degradation products provided the basis for a preliminary risk assessment, using an in silico evaluation, which showed that certain degradation products raised toxicological issues. These initial results were important as a basis for justifying and determining the threshold limits for impurity control in stability studies of the finished product by categorizing the degradation products to be specified and those which will not be specified.

In addition to these pre-formulation data, it was also crucial to seek to understand in detail the main degradation pathways of ONC201 to identify the influential parameters, thus opening the way to determining the appropriate levers to anticipate the instability of the active substance during the various pharmaceutical processes [4,5,6,7,8]. To address these issues, numerous methods combining separated or gathered knowledge acquired from computational studies and experiments have been published with the view to increase the specific understanding of each drug’s degradation pathways [9,10,11,12,13].

Therefore, the present study aimed to characterize in detail the degradation mechanisms of ONC201, with a view to determine the appropriate methods to minimize its degradation by oxidation and photolysis. Theoretical and complementary studies in the presence of antioxidants have been carried out, on the one hand, in order to support the hypotheses of the mechanisms involved and, on the other hand, to propose the conditions of their use to obtain the effective protection of ONC201 against oxidative degradation.

## 2. Materials and Methods

### 2.1. Reagents

ONC201 dihydrochloride (DiHCl) (batch #S22S02C27; purity > 99%) was purchased from MedKoo Biosciences (Morrisville, NC, USA). Analytical grade acetonitrile was obtained from Sigma-Aldrich (St Quentin-Fallavier, France). Ultrapure water was produced by the Direct-Q^®^ 3 UV system (Merck, Guyancourt, France). Hydrogen peroxide (H_2_O_2_) 30% *v*/*v* was supplied by Carlo Erba SDS (Val de Reuil, France), whereas hydrochloric acid and sodium hydroxide were obtained from Sigma-Aldrich (St Quentin-Fallavier, France).

The antioxidant agents used were ascorbic acid, N-acetyl cysteine, and sodium sulfite. The latter were supplied by Sigma-Aldrich (St Quentin-Fallavier, France).

### 2.2. Analytical Conditions

The solutions were analyzed via high-performance liquid chromatography (Agilent 1260 Infinity) coupled with diode array and mass spectrometry detection (6495 triple quadrupole, Agilent technology, Cheadle, UK). The stationary phase was a Phenomenex C18 column (250 × 4.6 mm; 5 µm). The mobile phase was a gradient combining 0.1% (*v*/*v*) formic acid added in both solvents: pure water and 0.1% formic acid (solvent A) and acetonitrile and 0.1% acid formic (solvent B). The gradient program was set as follows: 0 to 35 min, 95% A → 0%; 35–37 min, 95 → 5% B; 37–40 min, 95% A/5% B with a flow rate of 0.4 mL/min^−1^. The injection volume was 5 μL, and column temperature was set to 30 °C with a UV wavelength of 254 nm.

The UV spectra of ONC201 were recorded with a spectrophotometer UV 7 (Mettler Toledo; Viroflay, France); the pH of the studied solutions was fixed by the use of sodium hydroxide and chlorohydric acid.

### 2.3. Forced Degradation Test

Forced degradation studies were performed in accordance with ICH Q1A (R2) [14] and Q1B [15] guidelines. Stress studies on ONC201 were performed under photolytic, oxidative, hydrolytic and thermal conditions. The hydrolytic (acidic and basic), photolytic and oxidative stress studies were performed in solution phase, while the thermal stress was performed in solid phase.

The oxidation of ONC201 was studied by preparing an aqueous solution containing 0.3% H_2_O_2_ and 1 mg.mL^−1^ of ONC201 dihydrochloride (expressed in ONC201). This solution was stored at room temperature and the samples were analyzed after 0, 24, 48 and 72 h of exposure. To study the effect of antioxidants on ONC201 stability in the presence of 0.3% H_2_O_2_, ascorbic acid, N-acetylcysteine or sodium sulfite were added at a concentration of 0.5 mg.mL^−1^.

For photodegradation studies, ONC201 dihydrochloride aqueous solutions (1 mg.mL^−1^ expressed in ONC201) were exposed to simulated light using a Q-SUN Xe-1 xenon test chamber (Q-Lab Corporation, Saarbrücken, Germany) operating in window mode and samples were collected at 60 min, 3 h, 6 h, 24 h and 48 h. The light beam (300–800 nm) was set at an intensity of 1.50 W.m^−2^ at 450 nm.

### 2.4. Theorical Calculations

The main protonated isomer (pH = 4) and neutral isomer (pH = 8) of ONC201 were both subjected to conformational analysis employing the OPLS4 force field implemented in MacroModel program (MacroModel Schrödinger Release 2022-1: MacroModel; Schrödinger LLC: New York, NY, USA, 2022). Geometry optimizations were performed on each of the lowest energy conformers by the use of a B3LYP exchange–correlation functional with the D3 a posteriori correction [16] chosen, together with the 6-311G^++^** basis set. H-bond dissociation energies have been obtained using B3LYP combined to LACV3P^++^** basis set. The Molecular Electrostatic Potential (MEP), a descriptor providing information about the charge distribution that is useful for identifying molecular sites likely interacting with other molecules [10], was mapped as a function of electron density.

Maestro, MacroModel and Jaguar were used as implemented in the Schrödinger Materials Science Suite 2021-4. The results of the conformational analysis and of the coordinates of the two lowest energy conformers are provided in the Supplementary Materials (Figure S1 and Tables S1 and S2).

## 3. Results and Discussion

### 3.1. ONC201 Degradation under Light Exposure

#### 3.1.1. Proposed Degradation Mechanisms of ONC201 following Its Exposure to Light

To understand the mechanisms involved, as a crucial step to guide the subsequent stages of pharmaceutical development, we jointly used the capital of knowledge acquired from the structures of the photoproducts [3] and the data which make it possible to highlight the molecular reactivity of ONC201 based on the DFT.

**Figure 1:**
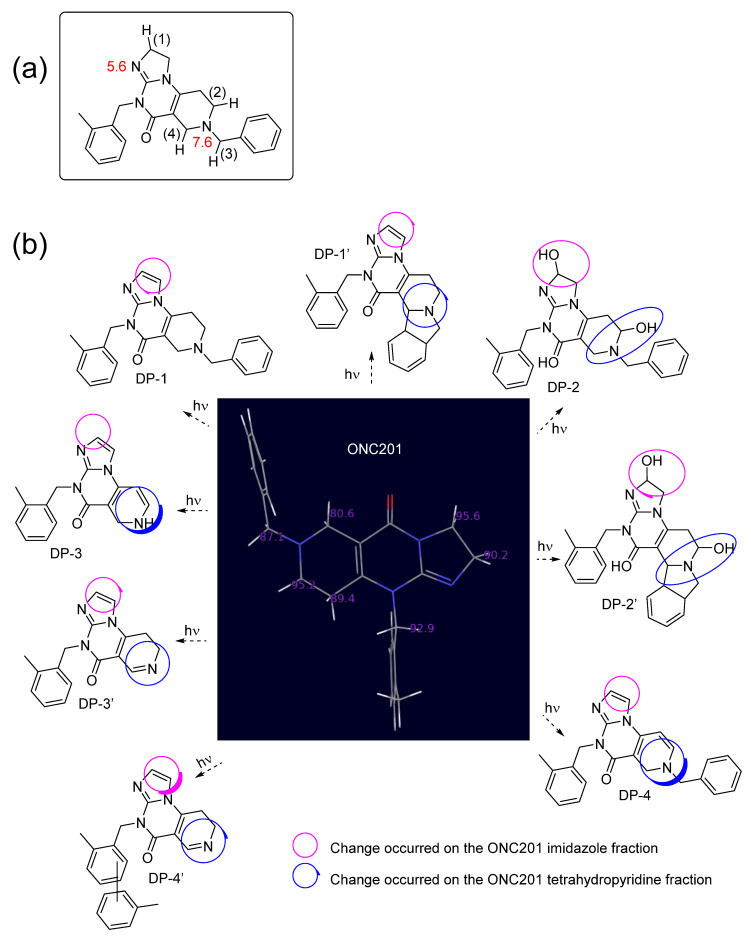
(**a**) Numbering of the main sites of the ONC201 molecule that were attacked under light stress conditions and a presentation of the pKa values (displayed in red); (**b**) ONC201 lowest BDE values at state S0 (figures displayed in purple) and ONC201 photodegradation products’ structures previously reported in article [3].

From the structures of the degradation products of ONC201 that have been identified via mass spectrometry (Figure 1) [3], it appears that two sites are primarily involved in degradation upon exposure to light, as the observed changes occurred in the vicinity of the imidazole imine nitrogen and/or the tetrahydropyridine nitrogen (positions (1), (2), (3) and (4), according to captions in Figure 1a). Indeed, the product arbitrarily named ONC201-DP-1 may exclusively be the result of the attacks in (1), whereas the other products reported in Figure 1b would be the result of both the attacks in (1) and those perpetrated in (2), (3) or (4). Attacks on the imidazole group result in the formation of additional unsaturation or the addition of an oxygen atom (Figure 1b), while attacks on the tetrahydropyridine group result in cyclization or cleavage, leading to the loss of the toluene part, as it is thereafter outlined.

It is very likely, from a mechanistic point of view, that an abstraction of the hydrogens present in the *alpha* position of the two nitrogen atoms could be the cause of the structures formed. Calculations based on molecular modeling techniques such as density functional theory (DFT) could be reliably used to predict the formation of the most stable radicals from hydrogen abstraction [17,18] and, therefore, help to strengthen the hypotheses of ONC201 degradation product structures. Specifically, the evaluation of bond dissociation energy (BDE) has been recognized as a good means of predicting and/or supporting the hypothesis of a radical abstraction oxidation mechanism, in particular if the corresponding BDE values are around or below 90 kcal mol^−1^ [19].

We performed these calculations considering both the protonation states and the excitation states (fundamental S0, singlet S1 and triplet T1) of the ONC201 molecule (Figure 1b and Table 1). It was found that the calculated BDE values are in perfect agreement with the results of the stress studies in that, irrespective of protonation and excited state, low C–H BDE values were found at sites (1), (2), (3) and (4), as shown in Figure 1a and Table 1.

Even in the ground state (S0), the BDE values of sites (1), (2), (3) and (4) of the ONC201 molecule are close to 90 kcal mol^−1^, regardless of its pH-dependent protonation state. Moreover, as these values drop sharply in the two excited states (Table 1), it can be assumed that the molecule then becomes particularly prone to chemical instability, leading to hydrogen departures by the homolytic breaking of the corresponding C–H bonds, thus initiating the process of drug substance degradation.

It should also be noted that this reduction, although notable, is much less dramatic at the acidic pH where the two nitrogen atoms of the tetrahydroimidazole and tetrahydropyridine groups are protonated (Table 1) according to their pKas (Figure 1a). This is in perfect coherence with the fact that the protonation of the amines or nitrogenous heterocycles makes it possible to improve the stability of the regions of the structure concerned [20].

**Figure 2:**
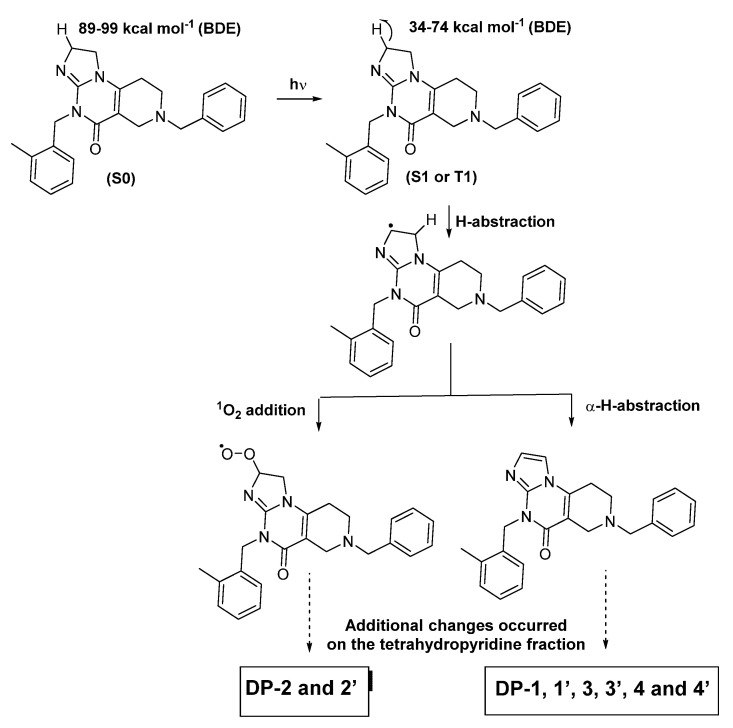
Formation of imidazole and of hydroxy-tetrahydroimidazole derivatives following the exposure of ONC201 to light.

**Figure 3:**
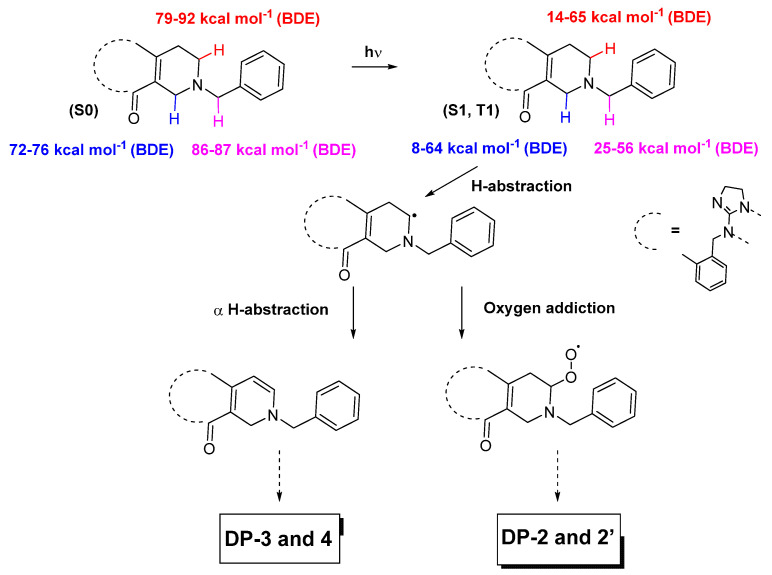
Formation of dihydropyridine and of hydroxy tetrahydropyridine derivatives following the exposure of ONC201 to light, following the homolytic break of the C–H bond at position (2) (Figure 1a).

**Figure 4:**
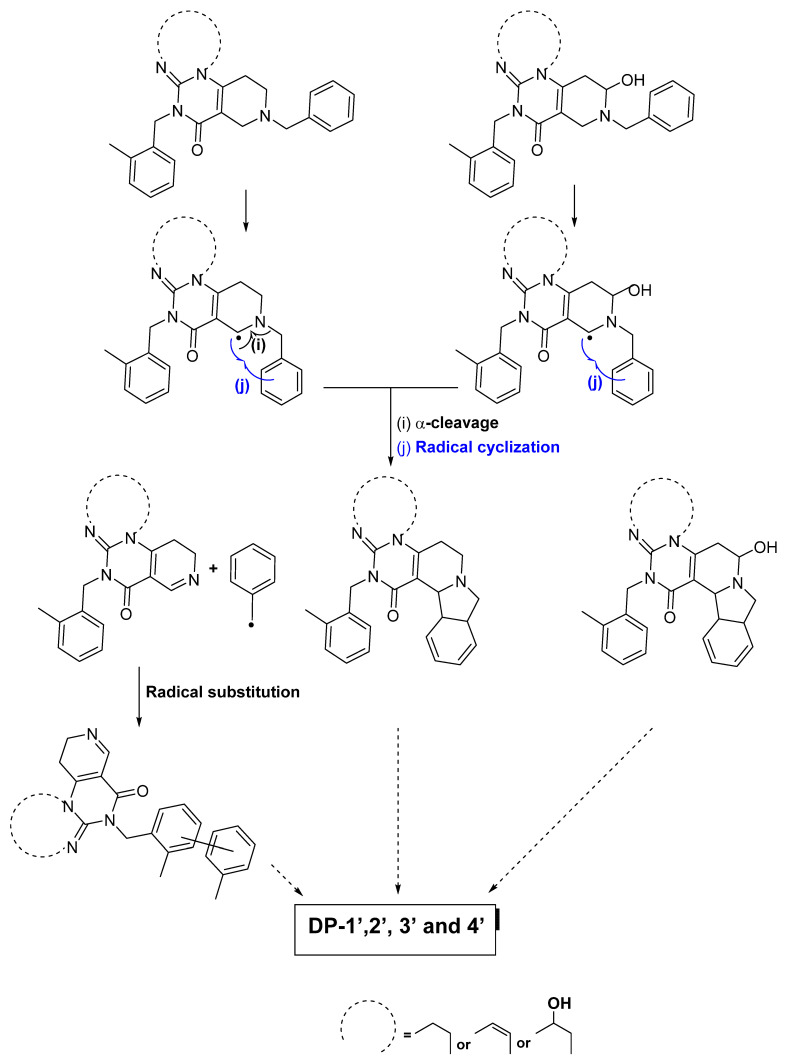
Formation of dihydropyridine and of hydroxy tetrahydropyridine derivatives following the exposure of ONC201 to light, following the homolytic break of the C–H bond at position (3) (Figure 1a).

Thanks to the convergence of previously published structural data [3] and these ab initio calculation data (Table 1), we were able to propose degradation schemes linking ONC201 to its degradation products (Figure 2, Figure 3 and Figure 4). As shown in Figure 1b, most degradation products detected during ONC201 stress studies exhibit an imidazole group instead of, initially, a tetrahydroimidazole group. Consecutively to the abstraction of the hydrogen from the C–H group in position (1) (Figure 1a), the process of radical propagation can take place, giving birth to other radical compounds. At the intramolecular level, it is quite conceivable that the π-bond of the imine function is displaced due to electron pooling. However, it appears that the departure of the hydrogen present in the *alpha* position from the radical site is a more rapid process, contributing to the termination reaction by leading to the formation of the imidazole group through the creation of additional unsaturation, as was the case for DP-1, 1′, 3, 3′, 4 and 4′ (Figure 1b and Figure 2). In competition with this termination process, the addition of singlet oxygen to the radical carbon would also have taken place as the latter is known to have fast kinetics and this helps to explain the formation in part of DP-2 and 2′ [3].

Simultaneously or sequentially, changes were also highlighted in the tetrahydropyridine moiety of the ONC201 molecule under the action of light. Likely, the mechanisms at stake would be like those that transformed the tetrahydroimidazole group into hydroxy tetrahydroimidazole and imidazole groups. Considering the data presented in Table 1, it appears that the C–H bonds in positions (2), (3) and (4) exhibit BDE values well below 90 kcal mol^−1^ in the S1 and T1 excited states, making them prone to homolytic breaks and leading to the formation of radical sites [19]. As a result of this, the same phenomena already described previously could also have taken place after the loss of the radical hydrogen from position (2) of the C–H group, namely the departure of a hydrogen atom located alpha from the radical site and the addition of singlet oxygen, given the actual formation of degradation products such as ONC201-DP-2, 2′, 3 and 4 (Figure 1b and Figure 3).

If there is hydrogen abstraction at the C–H group at position (3) (Figure 1a and Figure 4), in the absence of a hydrogen *alpha* to the radical site (linked to a sp^3^ carbon atom), an *alpha* cleavage leading to the departure of a radical toluene group or an intramolecular cyclization by radical substitution would have taken place. These mechanisms are in perfect agreement with the structures that have been identified for DP-1′, 2′, 3′ and 4′ (Figure 1b).

Finally, as for the formation of the 1,2-dihydropyridine-type derivative (DP-3), we hypothesized that the latter is related to the abstraction of the hydrogen from the C–H group in position (3). Following the formation of a radical site, the addition of oxygen can eventually lead to the formation of a function favorable to the heterolytic departure of a benzaldehyde group by N-dealkylation.

#### 3.1.2. Influence of pH on the Behavior of ONC201

While the convergence of previous approaches enabled us to propose photodegradation mechanisms for ONC201, we still had to remain cautious, as the molecule contains protonatable groups and the microspecies present at a given pH may not react in the same way to light. Indeed, the photodegradation of a compound by natural light strongly depends on its electronic absorption spectrum and that of its chemical speciation [21].

**Figure 5:**
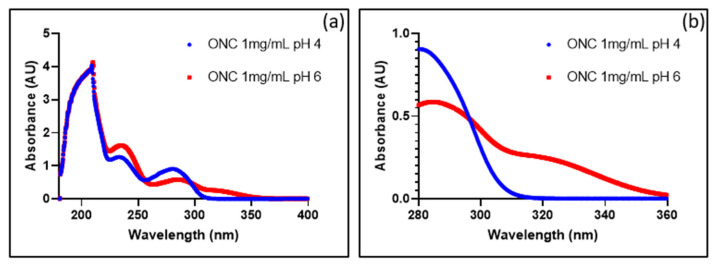
Effect of pH on the absorption spectrum of ONC201. (**a**) Spectrum from 180 nm to 400 nm; (**b**) zoom between 280 and 350 nm.

The UV/Visible spectra of ONC201 actually differ as a function of pH (Figure 5a), in the sense that its increase (pH 4 versus pH 6) translates spectrally into a bathochromic and hyperchromic effect in the UV–B region (280 to 320 nm, Figure 5b), suggesting that the molecule absorbs less natural light at an acidic pH and is therefore less sensitive to photolytic processes.

**Figure 6:**
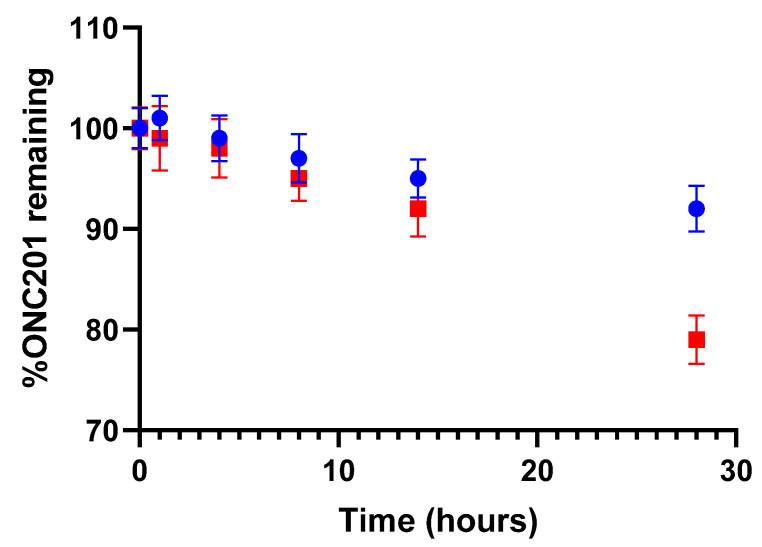
Effect of pH on the relative amount of ONC201 exposed to simulated light. Red points: solution at 1 mg.mL^−^^1^ adjusted to pH 6; blue points: solution at 1 mg.mL^−^^1^ without pH adjustment (pH 4).

On the experimental level, we quantitatively (Figure 6) and qualitatively confirm that we have obtained comparable chromatographic profiles of degradation products between pH 4 and pH 6. These results are in line with what was presented in Section 3.1.1, where it was shown that the lowest BDE values remain attributed to the same bonds regardless of the pH values ranging from 4 to 8, which explains a variation in the intensity of degradation but a similarity in the degradation products formed (Figure 6).

In other words, on a practical level, formulating a product based on ONC201 with a physiologically compatible acid pH adjustment would be a good way to stabilize the active substance, in addition to the precautions necessary to reduce exposure to natural light.

### 3.2. ONC201 Degradation in the Presence of Hydrogen Peroxide

#### 3.2.1. Proposed Degradation Mechanisms

As in the previous case, the guiding thread toward understanding the degradation process of ONC201 in the presence of an oxidizing agent (hydrogen peroxide) was the prior structural determination of its degradation products. For most of them, the degradation products contain one to several oxygen atoms in addition to what the parent molecule, ONC201, possessed [3].

Generally speaking, the degradation processes and kinetics induced by hydrogen peroxide are strongly affected by the pH of the media, as the pH value may favor either nucleophilic or electrophilic oxidation reactions [22]. The reaction of amines with hydrogen peroxide may slow dramatically when the nitrogen lone pair of electrons is protonated [22]. For this reason, the impact of pH value on degradation caused by the addition of H_2_O_2_ has also been taken into account in this assessment.

**Figure 7:**
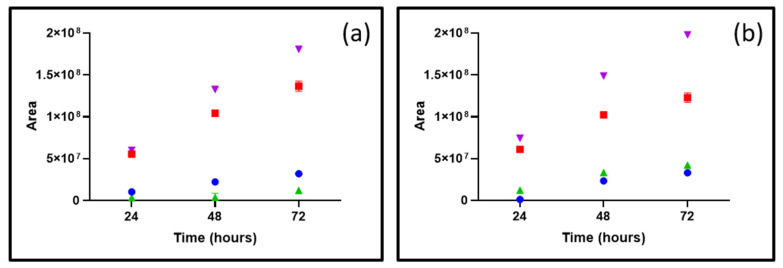
Signal intensities (expressed as peak areas) of chromatogram peaks as a function of pH ((**a**) for pH 4 and (**b**) for pH 6): (red points) case of DP-5; (blue points) case of DP-6; (green points) case of DP-7; (purple points) sum of areas of detected compounds other than ONC201.

**Figure 8:**
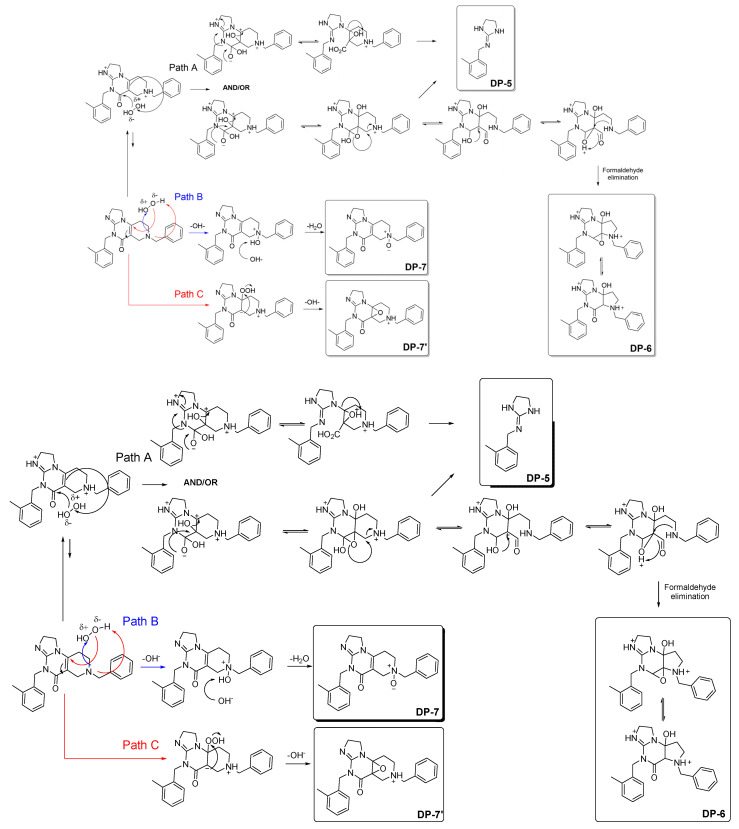
Proposed degradation pathways of ONC201 in aqueous media under the action of hydrogen peroxide, leading to the formation of oxidation products reported in the article [3].

Experimentally, the exposition of ONC201 to H_2_O_2_ gave rise to the quantitative formation of DP-5 and DP-6, which were also the first to appear after H_2_O_2_ addition (Figure 7). The formation of such degradation products de facto suggests that the sites concerned by reaction with H_2_O_2_ might correspond to the middle part of the molecule represented by the 2,3-dihydropyrimidin-4(1H)-one fraction. More specifically, the conjugation formed between ethylene and carbonyl moieties seems to make possible reciprocal interactions with the most electron-deficient oxygen atom, featuring the highest electron density of H_2_O_2_, such as that proposed in Figure 8 (path A). On this basis, we hypothesized the various rearrangement pathways favored by strong steric interactions to lead to the formation of the major degradation products already identified in our previous work [3].

In addition, we have shown that other types of oxidized products also coexist (Figure 7) [3]. Indeed, given the presence of numerous nitrogen functions such as a tertiary amine and several nitrogen heterocyclic functions, it was plausible to consider nucleophilic attacks on the most electron-deficient oxygen atom of the oxidizing agent used (H_2_O_2_) (Figure 8, path B), thus resulting in the formation of N-oxide derivatives [3]. According to the structures previously identified [3], the tertiary amine of the tetrahydropyridine moiety was mainly concerned by this reaction based upon the identified structures of DP-7 and DP-7′ (Figure 8, path B). Moreover, average local ionization energy (ALIE) calculations, using molecular modeling techniques by applying density functional theory (DFT), performed for the non-protonated form of ONC201 support these findings, in the sense that the tertiary amine nitrogen has the lowest ALIE value of all the nitrogen atoms present in the molecule [23] (Figure S1).

Eventually, electrophilic attacks could also occur, involving the anionic form of H_2_O_2_ present in trace amounts at acidic pH, but these reactions would still have been sufficiently quantitative to produce and allow for the detection of DP-7′ by LC-MS (Figure 8, path C) [24]. By carrying out the MESP topological study of the acid form of ONC201 (Figure S2), we noted the presence of a critical point (CP) located in the unsaturated region of the tetrahydropyridine group, which could make a nucleophilic attack possible, resulting in the formation of an epoxide function. This CP, which reflects an electron deficit, could be explained by the conjugation of the ethylene group with a carbonyl group acting as an electron-withdrawing group, making it more amenable to oxygen addition [25].

However, in both cases (Figure 8, paths B and C), when the pH of the reaction media is at 4, owing to the ONC201′s pKa values (5.6 and 7.6, Figure 1a), its protonated microspecies are present in a very large majority, which explains why these degradation pathways were limited (Figure 7a).

A decrease in two pH units significantly prevented the formation of DP-7 and DP7′ (around 70%). Contrarily to that, no subtle change in the amounts of DP-5 and DP-6 occurs under this condition. Thus, lowering the pH seems to help to decrease the degradation of ONC201 by limiting some of the degradation mechanisms at stake (Figure 7).

#### 3.2.2. Assessing the Effect of Antioxidants

Whatever the case, all the tests previously described in this article were carried out after dissolving ONC201 hydrochloride in water, giving rise directly to solutions with a pH close to 4. Solutions with pH 6 were obtained by adding a sufficient volume of 1 M sodium hydroxide.

**Figure 9:**
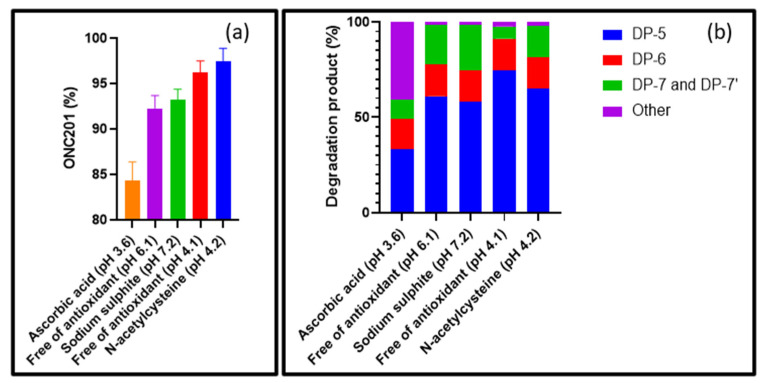
(**a**) Relative amount of ONC201 after exposure to 0.3% H_2_O_2_ for 72 h in the presence of antioxidants; (**b**) relative amounts of degradation products after exposure to 0.3% H_2_O_2_ for 72 h in the presence of antioxidants.

But, to test the action of antioxidants on ONC201 solutions exposed to H_2_O_2_, we measured the pH of the various reaction media without readjustment to gain a better understanding of the intrinsic role of these protective agents against H_2_O_2_ attacks (Table S1). We tested the effects of sodium sulfite, N-acetylcysteine and ascorbic acid at 0.5 mg.mL^−^^1^. We chose not to test the effect of tocopherol due to its poor solubility in water. The molar concentrations of the antioxidants were chosen so as to exceed that of H_2_O_2_. The percentage of ONC201 remaining after 72 h under each condition are gathered in Figure 9.

Compared with ONC201 alone in the presence of H_2_O_2_ (pH 4.1), we were able to confirm that the solution adjusted to pH 6.1 is more degraded (Figure 9a). In addition, its degradation product profile shows a relative increase in epoxide and N-oxide derivatives (Figure 9b), which helps to support the degradation pathways B and C proposed in Figure 8. What was unexpected, however, was what was observed with the addition of ascorbic acid or sodium sulfite. Indeed, both compounds having antioxidant properties we expected to obtain a protective effect, and, as a consequence, ONC201 should have been less degraded.

In the presence of sodium sulfite, we observe a profile of degradation products and a rate of degraded ONC201 that are entirely comparable with what was described for the previous case, i.e., the solution whose pH was adjusted to 6.1 with sodium hydroxide (Figure 9). It therefore appears that sodium sulfite has a negligible protective effect on ONC201 compared with acidic pH. Indeed, after dissolving sodium sulfite at a concentration of 0.5 mg.mL^−^^1^, the pH of the mixture was equal to 7.2 without any addition of sodium hydroxide.

The case associated with ascorbic acid is more troubling and complex. While it is known to be an effective pharmaceutical antioxidant in most cases [26], its addition not only led to a greater degradation of ONC201 compared to all the other cases (Figure 9), but was responsible for the formation of other hitherto unknown products not highlighted in any of the experiments we already carried out. The pH does not appear to be the cause, as it slightly decreased after the addition of the antioxidant. Although complementary experiments are needed to confirm this assumption, we postulate that these specific degradation processes may involve direct reactions with dehydroascorbic acid [27], formed by the oxidation of ascorbic acid. Further work is therefore needed to better understand these new data.

From all the antioxidants tested, N-acetylcysteine was shown to the a good candidate to prevent ONC201 from being degraded in the presence of H_2_O_2_ (Figure 9). As its addition did not induce a subtle change of the degradation profile and did not alter the pH value compared to the condition where no antioxidant is added, it seems that N-acetylcysteine has served to interact with H_2_O_2_ to prevent it from reacting with the active substance [28].

## 4. Conclusions

In addition to previously published data relating to studies aimed at characterizing the stability profile of ONC201 [3], in this study, we proposed the degradation mechanisms involved, following the exposure of this molecule to light and in an oxidative environment, conditions to which it is sensitive.

Although each step of the proposed mechanisms could not be confronted by the use of theoretical calculations, the combination of DFT-based and experimental results allowed us to better understand the role of pH in the stability of ONC201 and how these pH conditions are also important to allow certain antioxidants to act.

Based on these results, we have understood that the antioxidants should be chosen while bearing in mind two aspects. Precisely, the antioxidants must have a propensity to interact with hydrogen peroxide and oxygen and they must not be amenable to react with ONC201. We have shown that ascorbic acid could chemically interact with ONC201, resulting in the formation of compounds other than ONC201’s own degradation products.

The knowledge gained through this study will help to determine measures in view of mitigating ONC201 degradation and the related potential effects. This could be helpful for pharmaceutical development, in particular of an oral solution comprising ONC201.

## Figures and Tables

**Table 1:** C–H BDE values (kcal.mol^−1^) of the ONC201 structure as a function of the protonation (pH) and the excitation state (singlet S0, singlet S1 and triplet T1).

	BDE of Position (1) C–H Bond (Figure 1a)		BDE of Position (2) C–H Bond (Figure 1a)		BDE of Position (3) C–H Bond (Figure 1a)		BDE of Position (4) C–H Bond (Figure 1a)	
	[ONC201+2H]^2+^ (pH 4)	ONC201 (pH 8)	[ONC201+2H]^2+^ (pH 4)	ONC201 (pH 8)	[ONC201+2H]^2+^ (pH 4)	ONC201 (pH 8)	[ONC201+2H]^2+^ (pH 4)	ONC201 (pH 8)
**S0**	89	99	79	92	71	87	76	72
**S1**	74	48	65	41	56	38	64	21
**T1**	71	34	61	27	53	25	60	8

## Data Availability

Not applicable.

## Supplementary Materials

The following supporting information can be downloaded at: https://www.mdpi.com/article/10.3390/pharmaceutics15102371/s1. Table S1: Z matrix of the optimized conformer of [ONC201+2H]^2+^; Table S2: Z matrix of the optimized conformer of ONC201; Table S3: pH of aqueous solutions ONC201 in the presence of H_2_O_2_ (0.3%) and after pH adjustment or addition of ascorbic acid, sulphites and N-acetylcystein (final concentration: 0.5 mg.mL^−1^); Figure S1: results of the conformational analysis of [ONC201+2H]^2+^ (inset a) and of ONC201 (inset b); Figure S2: ALIE values (in kcal.mol^−1^) of the 4 nitrogen atoms of ONC201; Figure S3: Mapped electrostatic potential of study of the acid form of ONC201 and colour scale. Maximal and minimal MEP values are given in kcal.mol^−1^.
