# Supplementary material for: Molecular Mechanisms Involved in the Chemical Instability of ONC201 and Methods to Counter Its Degradation in Solution
Source: Pharmaceutics. 2023 Sep 22;15(10):2371. doi: 10.3390/pharmaceutics15102371 (PMC10609984; doi:10.3390/pharmaceutics15102371)
Supplement: Supplementary file 1 [file pharmaceutics-15-02371-s001.zip › pharmaceutics-2566040-supplementary.pdf]

Supplementary material

Molecular Mechanisms Involved in the Chemical Instability of ONC201 and Methods to Counter Its Degradation in Solution

Maxime Annereau [1,2], Marina Vignes [1,2], Lucas Denis [2], André Rieutord [2], François-Xavier Legrand [3], François Rioblanc [2], Muriel Paul [4,5], Jacques Grill [6,7], Philippe-Henri Secretan [8,*] and Bernard Do [1,4]

[1] Université Paris-Saclay, 91400 Orsay, France; maxime.annereau@gustaveroussy.fr (M.A.);
marina.vignes@gustaveroussy.fr (M.V.); bernard.do@aphp.fr (B.D.)

[2] Clinical Pharmacy Department, Gustave Roussy Cancer Campus, 94800 Villejuif, France; lucas.denis@gustaveroussy.fr (L.D.); andre.rieutord@gustaveroussy.fr (A.R.); francois.rioblanc@gustaveroussy.fr (F.R.)

[3] Université Paris-Saclay, CNRS, Institut Galien Paris-Saclay, 91400, Orsay, France; francois-xavier.legrand@universite-paris-saclay.fr

[4] Department of Pharmacy, Henri Mondor Hospital, AP-HP, 94000 Creteil, France; muriel.paul@aphp.fr

[5] EpidermE, Université Paris Est Creteil, 94000 Creteil, France

[6] Molecular Predictors and New Targets in Oncology, INSERM, Gustave Roussy, Université Paris-Saclay, 94800 Villejuif, France; jacques.grill@gustaveroussy.fr

[7] Département de Cancérologie de l'Enfant et de l'Adolescent, Gustave Roussy, Université Paris-Saclay, 94800 Villejuif, France

[8] Université Paris-Saclay, Matériaux et Santé, 91400 Orsay, France

* Correspondence: philippe-henri.secretan@universite-paris-saclay.fr

Table of contents

List of table 3

List of figures 3

List of table

Table S1: Z matrix of the optimized conformer of $[ONC201+2H]^{2+}$ 4
Table S2: Z matrix of the optimized conformer of ONC201 5
Table S3: pH of aqueous solutions ONC201 in the presence of H2O2 (0.3%) and after pH adjustment or addition of ascorbic acid, sulphites and N-acetylcystein (final concentration: 0.5 $mg.mL^{-1}$) 7

List of figures

Figure S1: results of the conformational analysis of $[ONC201+2H]^{2+}$ (inset a) and of ONC201 (inset b) 7
Figure S2: ALIE values (in $kcal.mol^{-1}$) of the 4 nitrogen atoms of ONC201 7
Figure S3: Mapped electrostatic potential of study of the acid form of ONC201 and colour scale. Maximal and minimal MEP values are given in $kcal.mol^{-1}$ 8

Table S1: Z matrix of the optimized conformer of $[ONC201+2H]^{2+}$

Atom	x-coordinate	y-coordinate	z-coordinate
O1	2.9968600000000	-4.5258890000000	8.8411600000000
N2	4.8202340000000	-4.3746860000000	12.4099330000000
N3	6.5645600000000	-2.2500660000000	9.3179020000000
N4	3.1300120000000	-5.2511010000000	11.0069600000000
C5	5.2861170000000	-3.5541840000000	11.4202640000000
C6	4.7128940000000	-3.5983600000000	10.1836220000000
C7	6.4410380000000	-2.6450470000000	11.7296590000000
C8	5.2225240000000	-2.7607590000000	9.0382750000000
C9	6.6163320000000	-1.5963680000000	10.6264480000000
C10	3.7373060000000	-5.2329430000000	12.2476960000000
C11	3.5759150000000	-4.4597790000000	9.9323970000000
C12	7.1198230000000	-1.4426820000000	8.2178930000000
C13	6.3798810000000	-0.1502960000000	7.9098990000000
C14	5.3464510000000	-0.1260770000000	6.9638280000000
C15	6.6744180000000	1.0287300000000	8.6080600000000
C16	4.6179040000000	1.0411540000000	6.7276410000000
C17	5.9478960000000	2.1978760000000	8.3770550000000
C18	4.9148620000000	2.2063100000000	7.4372930000000
C19	4.3666630000000	-5.6102180000000	14.3394490000000
N20	3.3984670000000	-5.9378150000000	13.2730480000000
C21	1.9940000000000	-6.1651380000000	10.7711700000000
C22	2.4126180000000	-7.4211920000000	10.0261620000000
C23	3.3888810000000	-8.2431510000000	10.6069860000000
C24	3.8337000000000	-9.4002140000000	9.9702890000000
C25	3.2926000000000	-9.7491680000000	8.7322280000000
C26	2.3148940000000	-8.9404730000000	8.1531150000000
C27	1.8575480000000	-7.7715580000000	8.7794210000000
C28	0.8014100000000	-6.9252090000000	8.1044920000000
C29	5.2254180000000	-4.4186750000000	13.8245170000000
H31	6.2826590000000	-2.1520610000000	12.6950830000000
H32	7.3501940000000	-3.2517100000000	11.8274050000000
H33	4.4978200000000	-1.9513790000000	8.8290030000000
H34	5.2579820000000	-3.3745320000000	8.1303010000000
H35	7.5878400000000	-1.1081700000000	10.7430170000000
H36	5.8451310000000	-0.8135830000000	10.7345400000000
H37	8.1605800000000	-1.2182360000000	8.4760090000000
H38	7.1334120000000	-2.0777120000000	7.3248070000000
H39	5.1145830000000	-1.0299230000000	6.4062700000000
H40	7.4817750000000	1.0312340000000	9.3354630000000
H41	3.8206760000000	1.0415240000000	5.9899140000000
H42	6.1897020000000	3.1024900000000	8.9275850000000
H43	4.3485240000000	3.1153350000000	7.2563290000000
H44	3.8460700000000	-5.3544150000000	15.2669340000000
H45	4.9936230000000	-6.4856240000000	14.5477970000000
H46	1.5938440000000	-6.4169420000000	11.7541080000000
H47	1.2396300000000	-5.6048610000000	10.2233660000000

H48	3.7991270000000	-7.9651000000000	11.5735660000000
H49	4.5925480000000	-10.0223060000000	10.4362800000000
H50	3.6277400000000	-10.6461670000000	8.2190870000000
H51	1.8963660000000	-9.2158870000000	7.1884350000000
H52	1.1576830000000	-5.8988080000000	7.9748660000000
H53	-0.1208510000000	-6.8819380000000	8.6945130000000
H54	0.5471930000000	-7.3324270000000	7.1231640000000
H57	6.3002390000000	-4.5867340000000	13.9147980000000
H56	4.9723840000000	-3.4735080000000	14.3153560000000

Table S2: Z matrix of the optimized conformer of ONC201

Atom	x-coordinate	y-coordinate	z-coordinate
O1	3.0907600000000	-4.6160760000000	8.7798460000000
N2	4.7668430000000	-4.3316670000000	12.3898320000000
N3	6.6585710000000	-2.3122240000000	9.2489550000000
N4	3.1349160000000	-5.2509930000000	10.9770080000000
C5	5.2907910000000	-3.5553630000000	11.3854520000000
C6	4.7599110000000	-3.6397590000000	10.1404060000000
C7	6.4601660000000	-2.6770570000000	11.7050940000000
C8	5.2842650000000	-2.8524030000000	8.9734990000000
C9	6.7112430000000	-1.6536020000000	10.6048140000000
C10	3.7015860000000	-5.1445150000000	12.1890040000000
C11	3.6289640000000	-4.4996920000000	9.8674970000000
C12	7.1766080000000	-1.4134030000000	8.1147560000000
C13	6.3886920000000	-0.1452320000000	7.9334570000000
C14	5.3177090000000	-0.1017320000000	7.0321530000000
C15	6.7274300000000	1.0103640000000	8.6471100000000
C16	4.5810740000000	1.0695830000000	6.8672310000000
C17	5.9905550000000	2.1810980000000	8.4835040000000
C18	4.9130560000000	2.2107230000000	7.5975730000000
C19	4.3256440000000	-5.4985360000000	14.3866410000000
N20	3.3475570000000	-5.7511420000000	13.3126600000000
C21	1.9918000000000	-6.1627480000000	10.7104890000000
C22	2.4248510000000	-7.4301370000000	9.9974990000000
C23	3.4620090000000	-8.1961220000000	10.5398590000000
C24	3.8857410000000	-9.3707670000000	9.9251560000000
C25	3.2610940000000	-9.7911670000000	8.7517170000000
C26	2.2257520000000	-9.0332910000000	8.2098700000000
C27	1.7891650000000	-7.8465890000000	8.8130900000000
C28	0.6715700000000	-7.0518960000000	8.1807550000000
C29	5.1273800000000	-4.3069790000000	13.8305880000000
H30	7.3034050000000	-3.1073240000000	9.2683270000000
H31	6.2882670000000	-2.1349560000000	12.6373700000000
H32	7.3427680000000	-3.3066410000000	11.8629170000000
H33	4.6364030000000	-2.0034670000000	8.7528590000000
H34	5.3461630000000	-3.4783110000000	8.0838760000000
H35	7.6943800000000	-1.2031850000000	10.7193300000000

H36	5.9530160000000	-0.8727710000000	10.6039400000000
H37	8.2157960000000	-1.2117000000000	8.3706730000000
H38	7.1491880000000	-2.0381290000000	7.2228280000000
H39	5.0629890000000	-0.9836100000000	6.4545470000000
H40	7.5714160000000	0.9983400000000	9.3271180000000
H41	3.7537410000000	1.0915110000000	6.1676490000000
H42	6.2604760000000	3.0694940000000	9.0424390000000
H43	4.3404100000000	3.1222570000000	7.4699570000000
H44	3.8145750000000	-5.2540840000000	15.3148140000000
H45	4.9480920000000	-6.3831190000000	14.5282130000000
H46	1.5042710000000	-6.3828860000000	11.6593240000000
H47	1.2836730000000	-5.5884290000000	10.1221170000000
H48	3.9532580000000	-7.8753550000000	11.4529140000000
H49	4.6922420000000	-9.9504560000000	10.3597930000000
H50	3.5784320000000	-10.7034520000000	8.2591970000000
H51	1.7456680000000	-9.3632730000000	7.2941880000000
H52	0.3360970000000	-7.5262300000000	7.2570090000000
H53	1.0019220000000	-6.0367090000000	7.9429450000000
H54	-0.1932020000000	-6.9708280000000	8.8468860000000
H57	6.1999930000000	-4.4302630000000	13.9549640000000
H56	4.8061030000000	-3.3558910000000	14.2560820000000
H58	2.6615220000000	-6.4876950000000	13.3714190000000

Table S3: pH of aqueous solutions ONC201 in the presence of H2O2 (0.3%) and after pH adjustment or addition of ascorbic acid, sulphites and N-acetylcystein (final concentration: 0.5 mg.mL^{-1})

Condition	Aqueous ONC201	Solution adjusted to pH=6	Ascorbic acid	Sulphite	N-acetylcystein
pH value	4.1	6.0	4.5	7.2	4.2

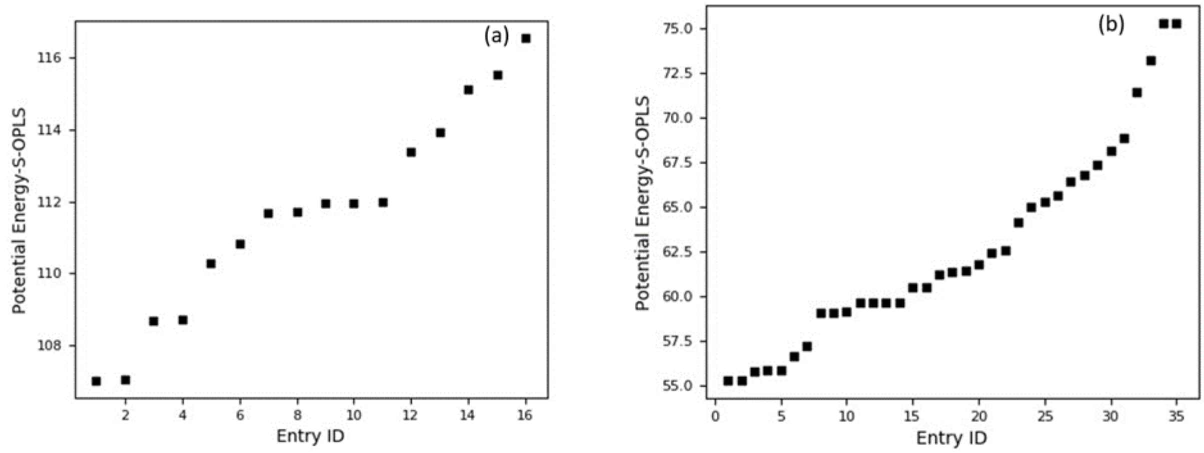

Figure S1: results of the conformational analysis of [ONC201+2H]$^{2+}$ (inset a) and of ONC201 (inset b)

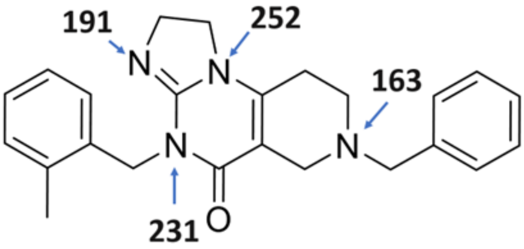

Figure S2: ALIE values (in kcal.mol^{-1}) of the 4 nitrogen atoms of ONC201

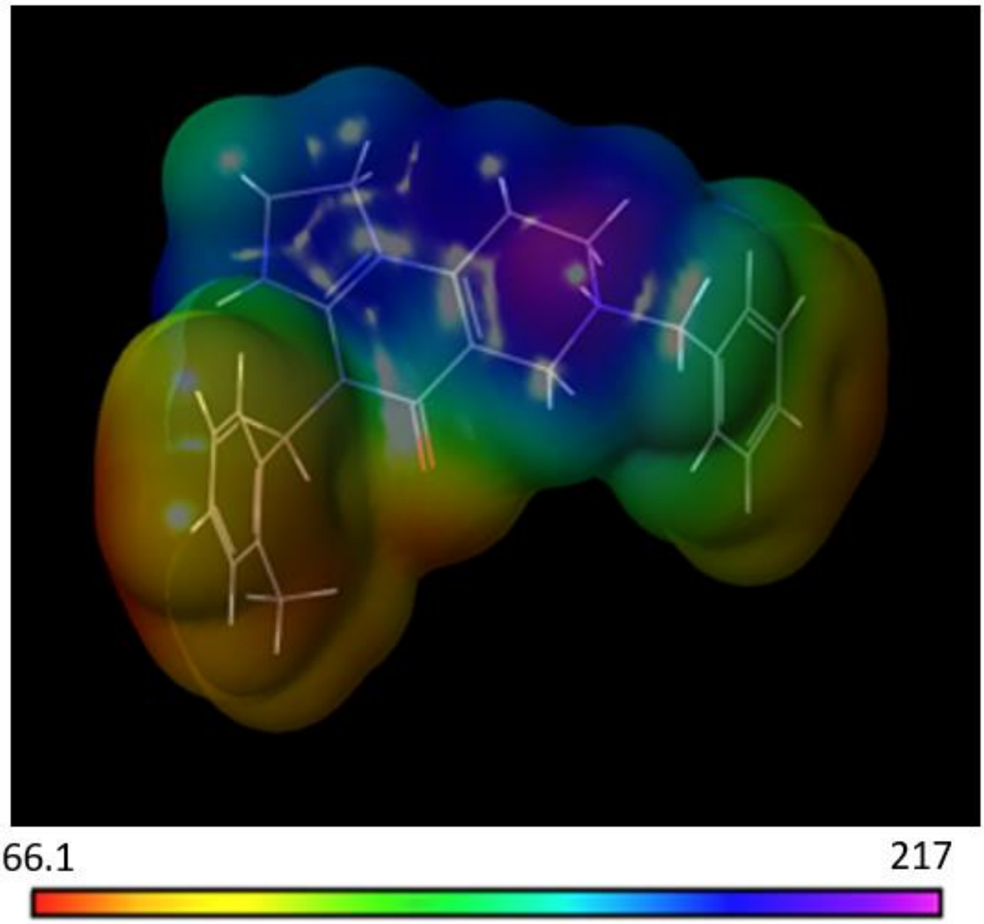

Figure S3: Mapped electrostatic potential of study of the acid form of ONC201 and colour scale. Maximal and minimal MEP values are given in kcal.mol^{-1}
